# Clinical assessment of neutrophil gelatinase-associated lipocalin as a potential diagnostic marker for neonatal sepsis: a prospective cohort study

**DOI:** 10.1080/07853890.2022.2091789

**Published:** 2022-06-30

**Authors:** Dina Midan, Fady El-Gendy, Dalia Abo ELAlla, Mayada Kotb

**Affiliations:** a Department of Pediatrics, Faculty of Medicine, Menoufia University, Menoufia, Egypt; b Department of Clinical Pathology, Faculty of Medicine, Menoufia University, Menoufia, Egypt; c Department of Pediatrics, Helwan University Hospital, Cairo, Egypt

**Keywords:** Neutrophil gelatinase-associated lipocalin, NGAL, diagnostic, neonates, sepsis

## Abstract

Sepsis is a life-threatening condition associated with high morbidity and mortality rates among neonates. Clinical diagnosis is limited due to the neonates’ unspecific signs and symptoms as well as the long time required to obtain the blood culture results. Consequently, there is an urgent need for new biomarkers to early diagnose neonatal sepsis. We aimed to evaluate Neutrophil Gelatinase-Associated Lipocalin (NGAL) diagnostic performance to detect neonatal sepsis. We enrolled 30 neonates with sepsis admitted to the neonatal intensive care units and 30 age- and sex-matched healthy neonates recruited from the neonatal outpatient clinic during their routine follow-up visits. We measured NGAL levels by sandwich enzyme-linked immunosorbent assay, the C-reactive protein (CRP) with nephelometry technique using BN II nephelometer, and the complete blood count by Mindray BC-6800 analysers. NGAL, CRP, TLC, haemoglobin, and platelet levels showed significant differences between cases and control (all *p* < .001). Of the 30 neonates with sepsis, 17 neonates (56.7%) survived. At 0 h, the NGAL level showed no statistically significant difference between the non-survivors and survivors’ groups; however, after 96 h, NGAL was significantly higher in the non-survivors group (*p* ˂ .001). Our diagnostic analysis showed that NGAL levels have strong discrimination power to early differentiate neonates with sepsis; at the 475.00 pg/ml cut-off value, NGAL showed both sensitivity and specificity of 100% with an area under curve of 100%. *Conclusion:* Our study suggests that NGAL could be a promising biomarker for neonatal sepsis detection. Further studies with larger sample sizes and survival analysis are warranted to confirm this finding and to clarify the efficacy of NGAL in survival prediction.

**Key findings**
- NGAL level was high in neonates with sepsis
- NGAL level was high in non-survived neonates
- NGAL could be a promising diagnostic marker for sepsis

## 1. Introduction

Despite the advances in life support management, infection is still a considerable cause of neonatal morbidity and mortality. Previous literature has documented factors that play a role in the high infection rate among neonates; these factors include prematurity, maternal genital colonisation, transplacental spread, traumatic delivery, and invasive practices such as arterial or umbilical catheterisation and underlying problems such as heart disease or hyaline membrane disease [1].

Sepsis diagnosis is confirmed upon positive microbiological culture results several days after empiric therapy initiation [2]. The typical use of antibiotic therapy for neonates having sepsis-like symptoms results in adverse drug effects, complications, and evolving of new resistant bacterial strains. Whereas the delay of antimicrobial therapy for sepsis cases results in severe complications such as systemic inflammatory responses and multiple organ dysfunctions leading to death [3,4].

Early accurate diagnosis of sepsis followed by proper management plays a crucial role in reducing infant sepsis-related mortality. Consequently, many studies evaluated hematological indices, procalcitonin, lipopolysaccharide-binding protein, acute phase reactants, cell surface receptors, cytokines as potential biomarkers for neonatal sepsis diagnosis. However, there is no current biomarker validated by multicenter clinical trials that could early detect neonatal sepsis [3].

Neutrophil gelatinase-associated lipocalin (NGAL) is a 24 kDa glycoprotein initially purified from kidney cell culture of murine infected with Simian virus 40. It is described as a biomarker for acute kidney injury. It is increased in serum, plasma, and urine after ischaemic kidney injury [5]. It is regarded as a specific marker of neutrophil activity. It is located in bone marrow cells as well as lung, bronchial, and colonic epithelial cells [6].

Increased NGAL concentrations have been demonstrated in the sera of patients with acute bacterial infections [6,7]. The level of NGAL was not elevated in healthy newborns at birth, while bacterial or fungal infections *in vivo* stimulate the rapid release of NGAL in newborns, even premature infants. Thus, NGAL may be an effective early marker of nosocomial neonatal infection [8]. We conducted this study to investigate the potential role of serum NGAL in the early diagnosis and prognosis of neonatal sepsis.

## 2. Methods

We conducted this prospective cohort study between January 2019 and June 2020 at the Menoufia University hospitals, Egypt. The study protocol was approved by the Menoufia Faculty of Medicine Committee for Medical Research Ethics (IRB: 4-2018PEDI32). The study was conducted in accordance with the Helsinki Declaration of 1964, as revised in 2013. Written informed consent was obtained from the guardian of each participant included in the study.

### 2.1. Eligibility criteria

The study group included all neonates (full-term and preterm neonates) with suspected neonatal sepsis diagnosed by Clinical sepsis score (CSS) and Hematological sepsis score (HSS)) [9,10] who later proved to have sepsis by blood culture. We excluded neonates with negative blood cultures and we didn’t include them as controls to avoid false negative sepsis results. Consequently, we chose neonates with no signs suspecting sepsis to serve as controls. Neonates with congenital anomalies, inborn errors of metabolism, perinatal asphyxia, infants of diabetic mothers and those with serum creatinine level >1.5 mg/dl were also excluded from the study.

### 2.2. Study process and evaluations

**Figure 1.**
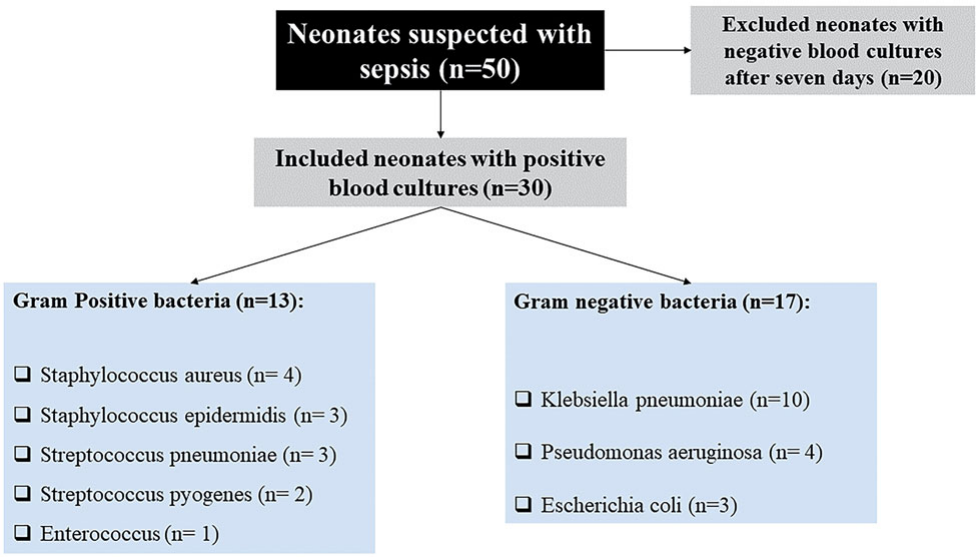
Cases selection criteria.

According to the sepsis occurrence, the included neonates were divided into two groups. The study group included 30 neonates with sepsis, confirmed by blood culture examination (Figure 1), admitted to the neonatal intensive care units (NICU). In comparison, the control group included 30 age- and sex-matched healthy neonates recruited from the neonatal outpatient clinic during their routine follow-up visits. All patients were subjected to a detailed history taking to detect sepsis risk factors as well as clinical and physical examination. The routine investigations included complete blood count (CBC), quantitative C-reactive protein (CRP) [11].

#### 2.2.1. Sample collection

Venous blood samples were collected from the controls during their visit to the outpatient clinic while seeking medical advice for simple skin or eye infections, nasal obstruction, jaundice, or clinical and laboratory preparations for operating circumcision in males. Samples were collected from the cases at the onset of the appearance of clinical signs suspecting that the neonate had sepsis (0 h) and again after 96 h from suspicion. Only suspected sepsis cases that were further proved to be sepsis by positive blood cultures were included in the study analysis. Then the samples were aseptically divided into two parts. The first part was collected into a plain vacutainer tube then centrifuged at 5000 RPM for 10 min. The collected sera were stored at −80 °C until the time of analysis of the NGAL and CRP. The second part was collected into EDTA-contained tubes to measure CBC within two days at maximum (were stored in the refrigerator at 4 °C).

#### 2.2.2. Biochemical and hematological analysis

NGAL concentrations were measured by sandwich enzyme-linked immunosorbent assay (ELISA) with commercially available kits (OmniKine™ Human NGAL ELISA Kit, Cat no. OK-0339). A monoclonal antibody specific for NGAL was pre-coated onto a microplate. Standards and samples were pipetted into the wells where the NGAL molecules bound to the immobilized antibody. An enzyme-linked monoclonal antibody specific for NGAL was added to the wells after washing away any unbound substances. A substrate solution was added to the wells and colour forms in proportion to NGAL bound in the initial step. The colour progression is ended, and the tension of the colour is measured. The dynamic range of the kits used in the analysis is 79–5000 pg/ml. We measured the serum CRP by nephelometry technique at 840 nm wavelength using a BN II nephelometer (Siemens) [12]. Besides, we measured the CBC using Mindray BC-6800 analysers [13].

### 2.3. Sample size

The sample size was calculated using Epi Info (2000) software at 95% confidence intervals and 80% power of the study. Based on the previous study conducted by Bhandare *et al.* 2018 [14], who reported that the mean NGAL in control and case groups were 77 (±64.77) and 158 (±79.35), the minimum sample size calculated was 42 neonates (21 neonates in each group).

### 2.4. Statistical analysis

We analysed the data using IBM SPSS advanced statistics version 25 (IBM Corp, NY, US). We presented the qualitative data in frequencies and percentages. We performed the Shapiro-Wilk test to determine the type of data distribution. Parametric data were presented as mean ± SD and non-parametric data as median and interquartile ranges (25th and 75th percentile).

Two group difference was measured using independent *t*-test or Mann–Whitney *U*-test for parametric and non-parametric data. Qualitative variables difference determined by Chi-square test. Pearson’s (for parametric data) and Spearman’s rho (for non-parametric data) correlation analysis were used including the entire cohort for 0 h and only septic neonates for 96 h results. Receiver operating characteristic (ROC) curve analysis was performed with using the Youden index to select the optimal cut-off values. The significant difference was considered when p-value <.05.

## 3. Results

### 3.1. Characteristics of the included population

Sixty neonates (29 male and 31 female) were included in our study. The neonates’ age, mode of delivery, gender distribution showed no significant difference between the study groups. The mean weight of neonates was 3.0 Kg in the control group versus 2.81 Kg in the sepsis group (*p* = .039). The median total leucocyte count (TLC) and CRP were statistically higher in the sepsis group than the control group (13.70 versus 8.15 × 10^3^/µl; *p*=.002 and 14.0 versus 4.0 mg/l; *p* ˂ .001). The sepsis group showed a statistically higher level of NGAL (1428 versus 239.17 pg/ml; *p* ˂ .001), as shown in Table 1. The median clinical sepsis score and median hematological sepsis score were 4.0 (IQR = 3.0 to 5.0) and 4.0 (IQR = 3.0 to 5.25). The median incubation period among the sepsis group patients was 28.0 days (IQR = 21.0 to 35.0).

**Table 1.** Demographic and clinical characteristics of the studied groups.

Variables	Cases (*n* = 30 neonates)	Control (*n* = 30 neonates)	P-value
Gestational age (weeks)	36 (35.0 to 37.0)	36 (35.7 to 37.0)	.557
Mode of delivery			
Cesarean section	16 (53.3%)	22 (73.3%)	.108
Normal vaginal delivery	14 (46.7%)	8 (26.7%)	
Gender			
Male	16 (53.3%)	13 (43.3%)	.438
Female	14 (46.7)	17 (56.7%)	–
Age (days)	1.0 (1.0 to 4.0)	2.0 (1.75 to 3.00)	.478
Weight (Kg)	2.81 ± 0.44	3.0 ± 0.28	.039^a^
Creatinine(mg/dl)	1.13 ± 0.32	0.6 ± 0.24	<.001^a^
Urea (mg/dl)	49.81 ± 6.18	23.8 ± 4.15	<.001^a^
Na+ (mEq/l)	135.81 ± 6.09	136.8 ± 6.81	<.001^a^
K+ (mEq/l)	4.54 ± 0.84	3.91 ± 0.38	<.001^a^
Total leucocyte count (×10^3^/µl)	13.70 (7.90 to 26.56)	8.15 (7.80 to 8.30)	.002^a^
Haemoglobin (g%)	12.8 ± 2.25	15.4 ± 0.89	˂.001^a^
Platelet (×10^3^/µl)	160..57 ± 74.86	257.13 ± 38.5	˂.001^a^
C-reactive protein, mg/l	14.0 (5.75 to 24.25)	4.0 (3 to 6)	˂.001^a^
NGAL (pg/ml)	1428 ± 440.8	239.17 ± 32.4	˂.001^a^
Non-survivors	13 (43.3%)	–	–
Complications			
Disseminated intravascular coagulation	4 (13.3%)	–	–
Heart failure	2 (6.6%)	–	–
Inter-ventricular haemorrhage	1 (3.3)	–	–
Necrotizing enterocolitis	3 (10%)	–	–
Pneumonia	5 (16.6)	–	–
Septic Shock	2 (6.6%)	–	–

Parametric data are expressed as mean ± SD, and non-parametric data are expressed as median (interquartile range). Qualitative data are expressed as frequency (percentage).

NGAL: Neutrophil Gelatinase-associated Lipocalin

^a^*p*< .05 *is significant*.

### 3.2. Characteristics of the survived versus non-survived cases

Of the 30 neonates with sepsis, 17 neonates (56.7%) survived. There was no statistically significant difference between both groups regarding NGAL at 0 h (*p*=.574). Haemoglobin and platelets were significantly higher among survivors. Moreover, TLC, CRP at 0 h, CRP after 96 h, and NGAL after 96 h were significantly higher in the non-survived neonates. Similarly, both clinical sepsis score and hematological sepsis score were higher in the non-survived neonates (all *p* ˂ .001), Table 2.

**Table 2.** Clinical characteristics among the survived and non-survived cases.

Variables	Survivors	Non-survivors	P-value
	(*n* = 17 neonates)	(*n* = 13 neonates)	
Total leucocyte count (×10)^3^/µl	8.2 (6.65 to 11.8)	26.8 (18.95 to 29.35)	˂.001^a^
Haemoglobin (g%)	14.25 ± 1.82	10.9 ± 0.99	˂.001^a^
Platelets (×10^3^/µl)	203.3 ± 57.5	104.67 ± 56.2	˂.001^a^
C-reactive protein at 0 h, mg/l	6.0 (5.0 to 11.5)	25.0 (17.5 to 29.5)	˂.001^a^
C-reactive protein after 96 h, mg/l	10.0 (3.0 to 31.5)	120 (58.5 to 163.5)	˂.001^a^
NGAL at 0 h, pg/ml	1382 ± 141	1489 ± 660	.574
NGAL after 96 h, pg/ml	600 (562.5 to 625.0)	3000 (1725 to 3175)	˂.001^a^
Clinical Sepsis Score (CSS)	3.0 (2.0 to 4.0)	6.0 (4.5 to 6.0)	˂.001^a^
Hematological Sepsis Score (HSS)	3.0 (2.0 to 4.0)	6.0 (4.5 to 6.0)	˂.001^a^

Data are expressed as mean ± SD for Gaussian data and median (interquartile range) for non-Gaussian data.

NGAL: Neutrophil Gelatinase-associated Lipocalin

^a^*p*< .05 *is significant*.

### 3.3. Correlation analysis between NGAL and other parameters

There were significant positive correlations between NGAL levels at 0 h as well as after 96 h and clinical sepsis scores (*r* = 0.458, *p*=.011 and *r* = 0.952, *p*<.001). Also, we detected a significant positive correlation between NGAL levels at 0 h as well as after 96 h and hematological sepsis score (*r* = 0.507, *p*<.004 and *r* = 0.917, *p*<.001), Table 3.

**Table 3.** Correlation analysis between the NGAL levels of the entire cohort at 0 h, septic cases at 96hs and other different parameters.

	NGAL at 0 h (pg/ml)		NGAL after 96 h (pg/ml)	
	Coefficient	P-value	Coefficient	P-value
Weight (Kg)	−0.072	.706	−0.453	.012^a^
Creatinine (mg/dl)	0.053	.512	0.061	.542
Urea (mg/dl)	0.081	.632	0.21	.534
Total leucocyte count (×10)^3^/µl	0.417	.022^a^	0.88	<.001^a^
Haemoglobin (g%)	−0.19	.316	−0.684	<.001^a^
Platelets (×10)^3^/µl	−0.258	.169	−0.682	<.001^a^
C-reactive protein at 0 h, mg/l	0.472	.009^a^	0.925	<.001^a^
C-reactive protein after 96 h, mg/l	–	–	0.848	<.001^a^
Clinical sepsis score	0.458	.011^a^	0.952	<.001^a^
Hematological Sepsis Score	0.507	<.004^a^	0.917	<.001^a^

NGAL: Neutrophil gelatinase-associated Lipocalin

^a^*p*< .05 *is significant*.

### 3.4. Efficacy of the studied parameters as diagnostic biomarkers for neonatal sepsis

**Figure 2.**
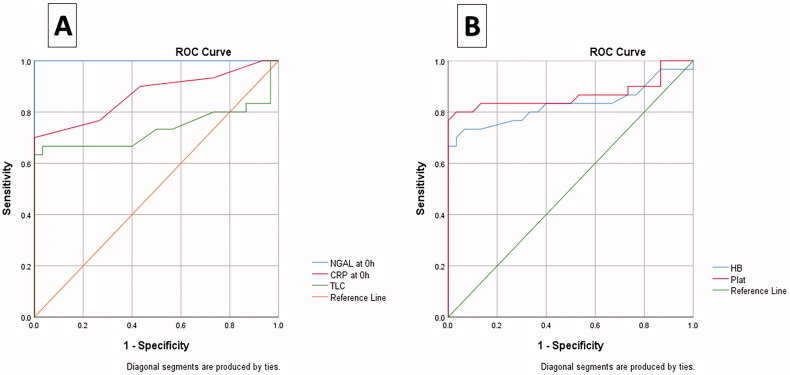
Receiver-operating characteristic (ROC) curve of (A) NGAL, CRP, and TLC; (B) haemoglobin and platelets for discrimination between septic patients and controls. CRP: C-reactive protein; NGAL: Neutrophil gelatinase-associated lipocalin; Plat: platelets; TLC: Total leucocyte count.

The usefulness of NGAL, as a diagnostic marker, compared with other sepsis markers, was tested through receiver-operating characteristic (ROC) curve analysis. NGAL was found to be better than the TLC and CRP. The optimum NGAL cut-off value to diagnose sepsis is 475.00. At ≥475.00 points, the NGAL showed both sensitivity and specificity of 100% with an area under the curve of 100%. Platelet count showed high sensitivity (80%) and specificity (96.7) to detect neonatal sepsis, as shown in Table 4 and Figure 2.

**Table 4.** Diagnostic performance of NGAL and other studied parameters as diagnostic markers for neonatal sepsis.

	Area under the curve	95% Confidence Interval		Cut-off		Sensitivity (%)	Specificity (%)
		Lower bound	Upper bound				
NGAL, pg/ml	1	1	1	475		100.00%	100.00%
C-reactive protein, mg/l	0.869	0.775	0.964	10.5		66.70%	100.00%
Total leucocyte count (×10)3/µl	0.736	0.592	0.879	10.55		63.30%	100.00%
Haemoglobin (g%)	0.829	0.713	0.945	14.35		73.30%	93.30%
Platelet (×10)3/µl	0.867	0.76	0.974	215		80.00%	96.70%

NGAL: Neutrophil gelatinase-associated Lipocalin.

## 4. Discussion

Routine neonatal clinical sepsis diagnosis mainly depends on blood culture, serum procalcitonin, and CRP levels. However, cultures delay the diagnosis by at least 48 h, and CRP and procalcitonin have low diagnostic efficacy [3,15]. Hence we evaluated a new parameter for the early diagnosis of neonatal sepsis. Our results elicited significant difference between cases and control regarding serum creatinine and urea (*p*<.001) and although NGAL is a known marker of kidney injury, we assume that its rise in our cases is attributed to sepsis itself as we basically recruited cases with serum creatinine level less than 1.5 mg/dl which is the known cut-off value for AKI in neonates [16]. We also studied cases with early onset sepsis in the first few days of life where the serum level of kidney function tests is considerably related to maternal kidney function tests. In patients without inflammation, the upsurge in plasma-NGAL may be a well-grounded marker to recognize the beginning of AKI. Regarding the patients undergoing mild to moderate inflammation, the diagnostic utility of plasma-NGAL for the detection of AKI may be confined. In these conditions, a mild or moderate increase in NGAL may associate more with inflammation rather than AKI by itself [17].

Our study revealed that septic neonates have significantly higher NGAL, CRP, TLC, and lower Hb and platelets than controls. 43.3% of the sepsis cases were not survived. Our diagnostic analysis showed that both NGAL and platelets have high sensitivity and specificity to discriminate septic neonates from controls. At the same time, CRP and TLC have high specificity but low sensitivity. Previous literature has suggested that NGAL upsurge is considered specific for sepsis [3,18–20]. Whereas, haemoglobin and platelet are decreased in sepsis patients due to decreased erythropoietin levels and the megakaryocytopoiesis process [21,22].

Comparing the survivors and non-survivors subgroups, the significant variations of NGAL, TLC, Hb, platelet, CRP, CSS, and HSS help to detect neonates that will need closer monitoring. We observed a positive correlation between NGAL and CRP, TLC, CSS, and HSS, and a negative correlation to Hb and platelets; similarly, these findings are in line with previous studies [23,24]. These variations are matched with the findings of Chang *et al.*, 2018 regarding NGAL, but on the other hand, they are not matched with prior studies regarding the TLC, CRP, HSS, and platelets, which may be because of the difference in the population studied and the eligibility criteria [18,25,26].

A recent meta-analysis conducted by Zhou *et al.* 2021 [5] on 28 studies showed that NGAL levels in serum and urine samples had pooled sensitivity of 83% and 87% and specificity of 79% and 84% to differentiate patients with sepsis-associated with acute kidney injury. This is consistent with our findings; however, our diagnostic analysis showed higher sensitivity and specificity at the 475.00 pg/ml cut-off value (both of 100% with an area under the curve of 100%). In our study, TLC and CRP showed high specificity (100%) but low sensitivities (63.3% and 66.7%, respectively). On the other hand, another study showed that they have low sensitivities (59.5% and 79.6%, respectively) and specificities (78.5% and 66%, respectively) to differentiate neonatal sepsis [27].

This research elucidated that platelets have high sensitivity (80%) and specificity (96.7) to differentiate septic neonates. For instance, thrombocytopenia in sepsis is caused by direct toxicity of platelets, suppression of megakaryocytes, or the presence of immune components on high levels of platelets [28]. While Hb has low sensitivity (73.3%) and high specificity (93.3%), the low levels of Hb in sepsis may be due to the destruction of red blood cells and high inflammatory response in patients with septic shock [29]. Our study has significant strength points being a prospective cohort study, and we investigated clinical characteristics among survived and non-survived cases besides investigating these characteristics among cases and controls. There are a few limitations regarding our study: (1) We did not compare the current parameters for the late-onset sepsis (2) other parameters such as procalcitonin were not measured.

In conclusion, our study suggests that NGAL could be a promising biomarker for neonatal sepsis detection. We recommend that further multicenter studies with larger sample sizes are warranted to investigate these findings and to investigate the survival in neonatal sepsis cases.

## Data Availability

Data are available upon reasonable request by contacting the corresponding author.

## Abbreviations

CBC: Complete blood count
CRP: C-reactive protein
CSS: Clinical sepsis score
Hb: Haemoglobin
HSS: Hematological sepsis score
NGAL: Neutrophil gelatinase-associated lipocalin
TLC: Total leucocyte count
